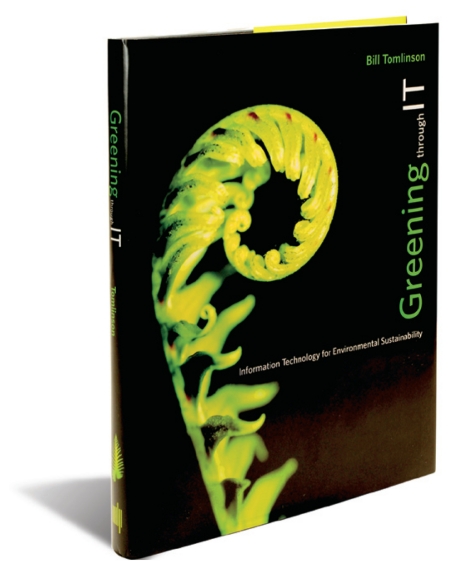# Greening through IT: Information Technology for Environmental Sustainability

**Published:** 2011-02

**Authors:** Eli Blevis

**Affiliations:** Eli Blevis is an associate professor of informatics in the Human–Computer Interaction Design program of the School of Informatics and Computing at Indiana University, Bloomington. His primary area of research is sustainable interaction design, within the confluence of human–computer interaction as it owes to the computing and cognitive sciences, and design as it owes to the reflection of design criticism and the practice of critical design. His research also engages design theory, digital photography, and studio-based learning.

The “through” in Bill Tomlinson’s title suggests a perspective that ascribes a fundamental role for information technology (IT) as an agency of environmental sustainability. The alternative—that IT also acts as agency of unsustainable practices—is also represented in Tomlinson’s description of the relationship between human behaviors and environmental conditions—especially climate change. Nonetheless, Tomlinson’s perspective lies in his words “cautiously optimistic,” seeing the role of IT in environmental issues and practices as a potentially net positive one included in the larger possibilities of humanity’s potential to act sensibly in all respects to climate change.

I would say that “cautious optimism” is indeed the theme. Tomlinson very aptly and with great integrity enumerates both environmentally positive and negative implications of the roles information technologies may play in mitigating climate change. He then seeks to find the cautiously optimistic line, appealing sometimes to very large-scale examples and sometimes to very personal experiences. Let me say at the outset that this is a book worth reading, indeed highly recommended.

For example, Tomlinson imaginatively conjectures two scenarios that might occur in this century to articulate the positive and negative limits of what is possible in human response to environmental concerns. Tomlinson’s illustrative “doomsday scenario” traces a possible course of future events from overpopulation and consequent overcultivation to an inevitable conclusion in which the “vast majority” of humanity perishes. Tomlinson’s illustrative “best-case scenario” is premised on both positive future technological development—the not quite plausible invention of “low-cost, sustainable, pollution-free energy”—and optimism about people’s behavioral willingness to adapt to sustainable lifestyles. Tomlinson’s “cautiously optimistic” middle is one in which more plausible technological advances and rapid, widespread behavioral change ensure “the survival of our descendants and the continuation of living things in general.”

As a reader and reviewer, I wonder about the logic and feasibility of finding a middle ground when faced with the prospect of reaching a tipping point with respect to climate change long before the necessary technological advances and sociopolitical will for behavioral change arrive. The notion of a “tipping point” is binary—once we reach a tipping point with respect to climate change, the feedback loop of negative effects spirals out of our control, and our efforts to reform are too late, by definition. Tomlinson is wholly optimistic about technology’s potential to mediate awareness about the need for urgent response: “If issues are presented to the people of the world in a compelling way, however, from both grassroots and governmental levels, it is possible that people may work together to come up with a viable future.”

Tomlinson’s cautious optimism describes an outward-looking view of IT’s role in the complex morass of all that is implicated in climate change and its mitigation. For an inward-looking perspective to describe what IT practitioners and researchers need to do differently or anew, Tomlinson introduces the theme of extended human-centered approach (EHCA) and extended human-centered computing (EHCC).

Within certain IT disciplines, the notion of human-centered computing (HCC) has been used to denote how IT may be applied in the service of individual human needs and interactivity, as opposed to the study of IT’s own internal operational theories and mechanisms. On the face of it, the idea of HCC seems like an intrinsically good thing, an incremental improvement over “user-centered design” (UCD). The term “user-centered design” has the unfortunate meaning that some individuals may be privileged over some others—it may be, for example, that designing mobile computing devices that are more easily discarded for newer devices than upgraded suits some users as a matter of convenience, but such a design comes at the expense of wasteful resource use and environmental damage for all of humanity and indeed the biosphere. The term “human-centered design” actually does little, if anything, to improve these semantics—it is better to refer to people as “humans” rather than as “users,” but the problem of privileging some groups and species over some others remains. Tomlinson points out that “there is a pervasive rhetoric associated with HCC that leads to short-term satisfaction of human users’ needs and desires.” The prevailing notion of HCC is therefore anathema to those who have been concerned with strategic—that is, long-term—design thinking about IT’s role in and accountability for global sustainability. The term “humanity-centered design” has been used to avoid the problems of the terms UCD and HCC, but even this term omits the notion of humankind’s accountability for its relationship to and compatible survival within the biosphere. Perhaps EHCC and EHCA can raise sustainability consciousness within IT disciplines effectively—should these terms achieve traction. Or perhaps a still stronger, sustainability-oriented term is needed that challenges the centrality of humankind’s base and perceived needs over balance within the natural world.

Regardless of whether the term EHCC raises consciousness enough, Tomlinson does use the notion to motivate an inward-looking view of IT that is informed by his outward-looking perspective on the social, political, and natural world. Framed by outward-looking notions of cautious optimism and inward-looking notions of EHCC, the book is reference quality for its inventory of how IT is implicated in sustainability and how sustainability is implicated in IT: for example, the use of IT systems to engineer smart power grids to make energy use more efficient and reduce GHG emissions; the possibility of using mobile IT technologies to enable small-scale agricultural production through more effective knowledge transfer; or the potential use of IT systems to enable product life-cycle assessment to minimize environmental impacts of manufacturing. Tomlinson appeals to areas such as evolutionary biology, competitive altruism, political and economic systems, energy, education, the classification of green IT systems, among others. He also provides three examples of green IT projects undertaken in his own research lab: the EcoRaft project, teaching schoolchildren about restoration ecology; the Trackulous project, promoting behavioral change by providing online interactive mechanisms of awareness about personal carbon footprints and other consequences of behaviors; the GreenScanner project, allowing consumers to make environmentally informed point-of-purchase product choices using a community-generated database of product reviews, mobile devices, and UPC code scanners.

I have focused on the two main outward- and inward-looking themes, but the book has other themes and much well-researched material that constitutes foundational knowledge about the present state of IT with respect to sustainability. This book is a “must read,” and it represents a more sophisticated, thoughtful view than most of what is presently chronicled under the banner of sustainability in the context of IT, even within the book’s most closely associated discipline of human–computer interaction.

To reach beyond the issues that Tomlinson enumerates entails a perhaps still more sobering, less positivist account of the need for both adaptation to and mitigation of the effects of climate change. Tomlinson’s cautious optimism and willingness to extend HCC represent to some degree a view of sustainability as an achievable goal in and of itself—one in which information technologies play a key role. Some might argue that we have already failed at the plan to avoid climate change by any means—technological, social, political, or otherwise—and that we might be better advised to focus on adapting as well as possible to a world in which the social and political will to achieve sustainability and avoid the consequences of climate change may arrive too late to succeed. This less optimistic possibility is also represented here and there in Tomlinson’s book. Let us hope that Tomlinson’s optimistic side prevails.

## Figures and Tables

**Figure:**